# Enhancing the one health initiative by using whole genome sequencing to monitor antimicrobial resistance of animal pathogens: Vet-LIRN collaborative project with veterinary diagnostic laboratories in United States and Canada

**DOI:** 10.1186/s12917-019-1864-2

**Published:** 2019-05-06

**Authors:** Olgica Ceric, Gregory H. Tyson, Laura B. Goodman, Patrick K. Mitchell, Yan Zhang, Melanie Prarat, Jing Cui, Laura Peak, Joy Scaria, Linto Antony, Milton Thomas, Sarah M. Nemser, Renee Anderson, Anil J. Thachil, Rebecca J. Franklin-Guild, Durda Slavic, Yugendar R. Bommineni, Shipra Mohan, Susan Sanchez, Rebecca Wilkes, Orhan Sahin, G. Kenitra Hendrix, Brian Lubbers, Deborah Reed, Tracie Jenkins, Alma Roy, Daniel Paulsen, Rinosh Mani, Karen Olsen, Lanny Pace, Martha Pulido, Megan Jacob, Brett T. Webb, Sarmila Dasgupta, Amar Patil, Akhilesh Ramachandran, Deepanker Tewari, Nagaraja Thirumalapura, Donna J. Kelly, Shelley C. Rankin, Sara D. Lawhon, Jing Wu, Claire R. Burbick, Renate Reimschuessel

**Affiliations:** 1 Veterinary Laboratory Investigation and Response Network (Vet-LIRN), Center for Veterinary Medicine, United States Food and Drug Administration, 8401 Muirkirk Rd, Laurel, MD 20708 USA; 2 Population Medicine & Diagnostic Sciences, Cornell University, Ithaca, New York USA; 3 Ohio Department of Agriculture, Ohio Animal Disease Diagnostic Laboratory, Reynoldsburg, OH USA; 4 School of Veterinary Medicine, Louisiana State University, Baton Rouge, LA USA; 5 Veterinary and Biomedical Sciences, South Dakota State University, Brookings, SD USA; 6 Animal Health Laboratory, University of Guelph, Guelph, Canada; 7 Florida Department of Agriculture and Consumer Services, Bronson Animal Disease Diagnostic Laboratory, Kissimmee, FL USA; 8 Athens Veterinary Diagnostic Laboratory, Department of Infectious Diseases, College of Veterinary Medicine, The University of Georgia, Athens, GA USA; 9 Tifton Veterinary Diagnostic and Investigational Laboratory, The University of Georgia, Tifton, GA USA; 10 Department of Veterinary Diagnostic and Production Animal Medicine, Iowa State University, Ames, IA USA; 11 Animal Disease Diagnostic Laboratory, Purdue University, West Lafayette, IN USA; 12 Veterinary Diagnostic Laboratory, Kansas State University, Manhattan, KS USA; 13 Breathitt Veterinary Center, Murray State University, Murray, KY USA; 14 Veterinary Diagnostic Laboratory, Michigan State University, East Lansing, MI USA; 15 Veterinary Diagnostic Laboratory, University of Minnesota, St. Paul, MN USA; 16 Veterinary Research and Diagnostic Lab System, Mississippi State University, Starkville, MS USA; 17 North Carolina State University College of Veterinary Medicine, Raleigh, NC USA; 18 Veterinary Diagnostic Laboratory, North Dakota State University, Fargo, ND USA; 19 New Jersey Department of Agriculture, Animal Health Diagnostic Laboratory, Ewing Township, NJ USA; 20 Oklahoma Animal Disease Diagnostic Laboratory, Oklahoma State University, Stillwater, OK USA; 21 Pennsylvania Department of Agriculture, Pennsylvania Veterinary Laboratory, Harrisburg, PA USA; 22 Pennsylvania Animal Diagnostic Laboratory, New Bolton Center, University of Pennsylvania, Kenneth Square, PA USA; 23 School of Veterinary Medicine, The Ryan Veterinary Hospital Clinical Microbiology Laboratory, University of Pennsylvania, Philadelphia, PA USA; 24 Texas A&M University, College Station, TX USA; 25 College of Veterinary Medicine, Washington Animal Disease Diagnostic Laboratory, Washington State University, Pullman, WA USA

**Keywords:** Antimicrobial resistance, Surveillance, One health, Whole-genome sequencing

## Abstract

**Background:**

Antimicrobial resistance (AMR) of bacterial pathogens is an emerging public health threat. This threat extends to pets as it also compromises our ability to treat their infections. Surveillance programs in the United States have traditionally focused on collecting data from food animals, foods, and people. The Veterinary Laboratory Investigation and Response Network (Vet-LIRN), a national network of 45 veterinary diagnostic laboratories, tested the antimicrobial susceptibility of clinically relevant bacterial isolates from animals, with companion animal species represented for the first time in a monitoring program. During 2017, we systematically collected and tested 1968 isolates. To identify genetic determinants associated with AMR and the potential genetic relatedness of animal and human strains, whole genome sequencing (WGS) was performed on 192 isolates: 69 *Salmonella enterica* (all animal sources), 63 *Escherichia coli* (dogs), and 60 *Staphylococcus pseudintermedius* (dogs).

**Results:**

We found that most *Salmonella* isolates (46/69, 67%) had no known resistance genes. Several isolates from both food and companion animals, however, showed genetic relatedness to isolates from humans. For pathogenic *E. coli*, no resistance genes were identified in 60% (38/63) of the isolates. Diverse resistance patterns were observed, and one of the isolates had predicted resistance to fluoroquinolones and cephalosporins, important antibiotics in human and veterinary medicine. For *S. pseudintermedius*, we observed a bimodal distribution of resistance genes, with some isolates having a diverse array of resistance mechanisms, including the *mecA* gene (19/60, 32%).

**Conclusion:**

The findings from this study highlight the critical importance of veterinary diagnostic laboratory data as part of any national antimicrobial resistance surveillance program. The finding of some highly resistant bacteria from companion animals, and the observation of isolates related to those isolated from humans demonstrates the public health significance of incorporating companion animal data into surveillance systems. Vet-LIRN will continue to build the infrastructure to collect the data necessary to perform surveillance of resistant bacteria as part of fulfilling its mission to advance human and animal health. A One Health approach to AMR surveillance programs is crucial and must include data from humans, animals, and environmental sources to be effective.

**Electronic supplementary material:**

The online version of this article (10.1186/s12917-019-1864-2) contains supplementary material, which is available to authorized users.

## Background

Antimicrobial resistance (AMR) is a global public health threat, and in the United States alone at least 23,000 people die each year due to resistant bacterial infections [1]. It is also a One Health issue because AMR emergence in bacteria from humans, animals, or the environment can impact the health of the others [2]. As such, it is critical to identify and characterize emerging AMR threats in each of these reservoirs so that integrated control policies may be developed.

Since 1996, the U.S. Centers for Disease Control and Prevention (CDC), the Food and Drug Administration (FDA), and the U.S. Department of Agriculture (USDA) have successfully monitored the development of AMR in foodborne pathogens through the National Antimicrobial Resistance Monitoring System (NARMS). This program is an integrated surveillance system that monitors the presence and resistance of foodborne pathogens from healthy food animals, retail meats, and human patients. These data provide valuable information on how AMR in the food supply may affect human health [3]. However, until this study, there was no systematic data collection of bacterial isolates from companion animals in the US, or among other integrated surveillance systems such as those in Denmark and Canada [4, 5].

It is essential that data from animal pathogens collected by veterinary diagnostic laboratories be incorporated into AMR surveillance activities as part of the One Health framework. These data, from bacterial pathogens of clinically ill veterinary patients, are an important addition to other surveillance programs that look at bacteria from healthy farm animals, foods and ill humans. Including veterinary pathogens in AMR surveillance will directly assist the veterinary profession treating our companion animals and will indirectly enhance our understanding of the epidemiology of AMR. The data from such studies can also be used to develop antimicrobial use (AMU) guidelines to educate veterinarians on the principles of good antimicrobial stewardship in their daily practice. Since the health of humans and animals are intricately linked, this data source is one of the critical components of One Health surveillance [6].

In March of 2015, the United States National Action Plan for Combating Antibiotic-Resistant Bacteria (CARB) was released to guide government, public heath, healthcare, and veterinary partners in addressing the AMR threat [7]. The National Action Plan specifically charged the FDA Veterinary Laboratory Investigation and Response Network (Vet-LIRN) with developing, expanding, and maintaining capacity in veterinary and food safety laboratories to conduct standardized antimicrobial susceptibility testing (AST) and characterize priority animal pathogens through whole genome sequencing (WGS). Other partners in this effort include the USDA’s National Animal Health Laboratory Network (NAHLN) and NARMS.

In order to address the tasks outlined by the CARB initiative, representatives from FDA, USDA and the American Association of Veterinary Laboratory Diagnosticians (AAVLD) formed the AAVLD Antimicrobial Resistance Working Group (Working Group). The Working Group conducted a survey among veterinary diagnostic laboratories in the U.S. [8] to identify which bacteria are commonly obtained in clinical laboratories and to develop a priority list of pathogens for surveillance. Using the recommendations of the Working Group [8] the FDA Center for Veterinary Medicine (CVM) developed a pilot program to evaluate the feasibility of using veterinary diagnostic laboratories in the Vet-LIRN network to monitor the antimicrobial susceptibility of selected veterinary pathogens. The four key objectives of the pilot project were to develop the laboratory infrastructure for a collaborative project with multiple participating veterinary diagnostic laboratories, to confirm laboratory proficiency for AST and WGS, to develop technology for sharing data within the network and to make the information publicly available.

The Working Group recommended *Escherichia coli*, *Salmonella enterica*, and *Staphylococcus pseudintermedius* for resistance monitoring based on frequency of culture, importance of the pathogen in clinical practice and availability of standardized AST methods for the pathogens. Dogs comprise the majority of clinical diagnostic veterinary isolates, and scientific reports also suggest that pathogens may transmit between humans and companion animals [9–11]. As a result, Vet-LIRN collected both *S. pseudintermedius* and *E. coli* specifically from dogs, where each bacterium frequently causes self-limiting infections. *Salmonella* was collected from all hosts, where it can cause gastrointestinal or in some cases systemic infections in a variety of animal species.

The Vet-LIRN program funded new AST testing equipment for multiple laboratories since the launch of the CARB initiative. Additionally, support from Vet-LIRN has rapidly increased the capacity for standardized WGS in U.S. veterinary diagnostic laboratories by providing state-of-the-art equipment and training. However, the Working Group survey [8] also noted considerable variation in AST methods, inhibiting direct comparison between labs. As genotypic markers of resistance identified by WGS match phenotypic measures approximately 99% of the time for *S. enterica* and *E. coli*, WGS can serve as reasonable proxy for traditional AST methods, circumventing the limitations noted in the survey [12–14]. Additionally, WGS can provide information on the potential transmissibility of resistance on mobile elements and the relatedness of isolates to those causing human illness [15].

Here we describe the data collection and WGS results from 2017, the first year of the pilot program, which included *E. coli* and *S. pseudintermedius* from dogs and *S. enterica* from any host animal. Specifically, we sought to assess the prevalence of antimicrobial resistance genes (ARGs) in our study population and the genetic backgrounds in which these ARGs are present.

## Results

A total of 1968 isolates (691 *E. coli,* 691 *S. pseudintermedius*, and 586 *S. enterica*) were collected, 200 of which were sequenced (68 *E. coli*, 71 *S. enterica*, and 61 *S. pseudintermedius*). The anatomical sites from which these isolates were collected is shown in Table 1.

**Table 1:** Anatomical site from which pathogen was isolated

Anatomical site:	*E. coli*	*S. pseudintermedius*	*S. enterica*
abscess	0	3	0
air sac	0	0	1
aspirate swab	0	1	0
bladder	2	0	0
brain	0	1	0
crop	0	0	1
ear	5	10	0
gall bladder	1	0	0
GI/fecal	5	1	47
heart	0	0	1
joint	0	1	2
kidney	0	0	1
liver	0	0	5
lung	6	3	7
lymph node	0	0	1
nasal swab	0	1	0
prostatic wash fluid	1	0	0
skin	6	26	0
unspecified/swab	0	1	0
unspecified/tissue	3	5	5
urine	35	5	0
uterus	1	0	0
vaginal swab	1	0	0
wound	2	3	0
Total	68	61	71

Eight isolates were excluded from the initial set of 200 sequenced isolates. Four *E. coli* isolates were excluded because they were collected from non-canine hosts (ECOL-17-VL-LA-KS-0031, ECOL-17-VL-LA-KS-0009, ECOL-17-VL-LA-KS-0046, and ECOL-17-VL-SD-NC-0028). One *E. coli* sequence (ECOL-17-VL-SD-OK-0009) and one *S. enterica* isolate (SAL-17-VL-LA-ND-0006) were excluded because of unusually long total assembly lengths. These two sequences also had > 20% of their assembly length classified as to a different species (*S. enterica* and *Enterobacter cancerogenus*, respectively). Two other isolates, one *Salmonella* (SAL-17-VL-SD-NC-0013) and one *S. pseudintermedius* (SPSE-17-VL-LA-KY-0018) were excluded after the majority of the assembly length was classified as a different species (*Citrobacter braakii* and *S. schleiferi*, respectively). The final dataset consisted of 63 *E. coli*, 69 *S. enterica*, and 60 *S. pseudintermedius* sequences.

### E. coli

**Fig. 1:**
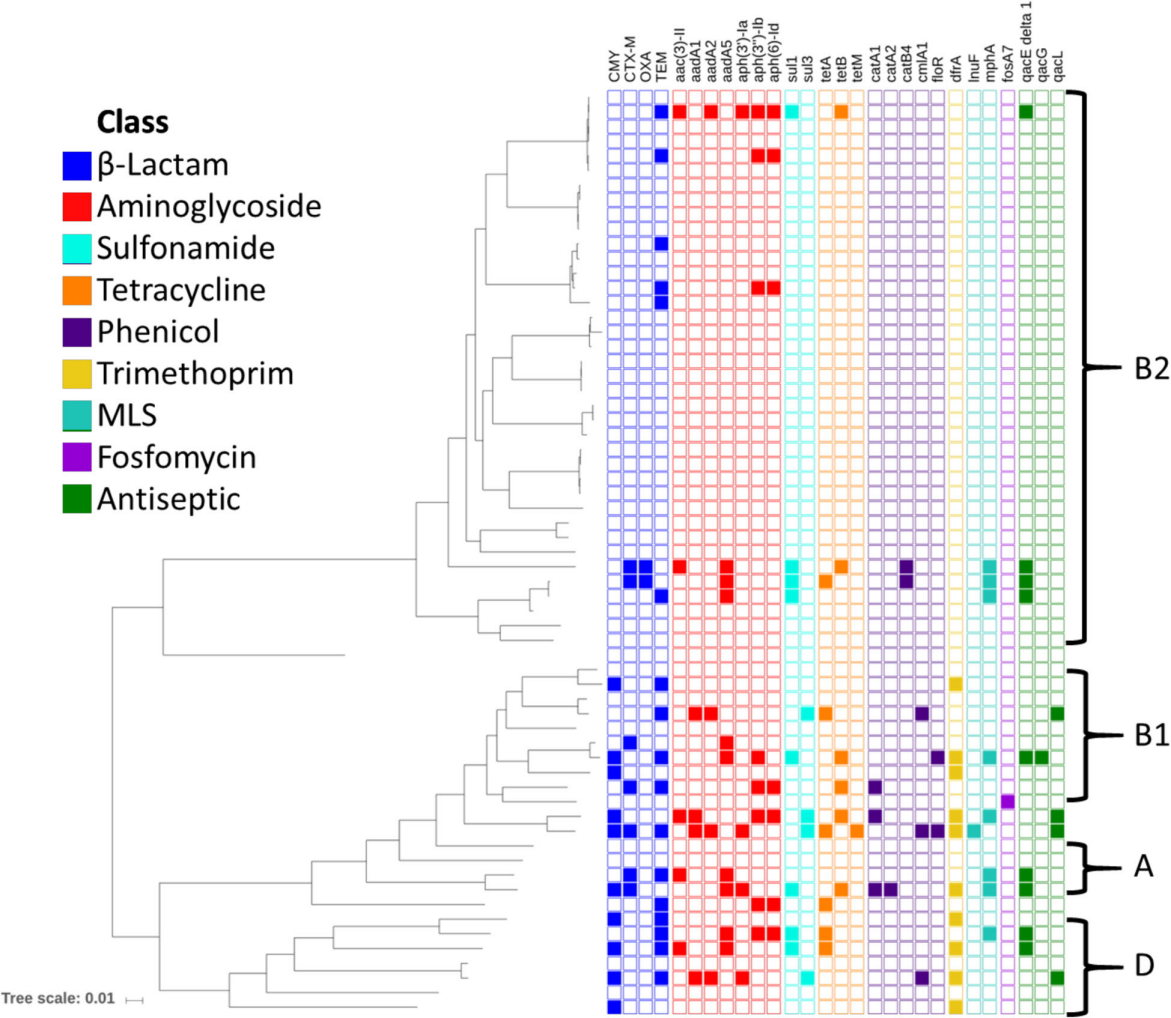
Phylogeny and Antimicrobial Resistance Gene Predictions in *E. coli.* Midpoint-rooted core genome phylogenetic tree of *E. coli* isolates with ARG predictions. Each column corresponds to the ARG listed along the top, with colors corresponding to the antibiotic class to which that gene confers resistance. A filled box indicates the detection of that gene

No resistance genes were identified in 60% percent of the *E. coli* isolates. The majority of *E. coli* isolates (38/63, 60%) were classified as phylogroup B2, including one that matched the atypical profile reported by Mendonça and colleagues [16], but clustered with other B2 isolates in the phylogeny (*n* = 38) [Fig. 1]. Fewer ARG were detected in phylogroup B2 isolates (median: 0, interquartile range: 0–0) as compared to those that belonged to other phylogroups (median: 2, interquartile range: 0–8). Fifteen isolates (24%) were predicted to be resistant to cephalosporins, conferred by *bla*_CMY_ and *bla*_CTX-M_ genes. Eight isolates also possessed *mphA* genes predicted to confer macrolide resistance. Fourteen isolates had *gyrA* amino acid substitutions expected to confer fluoroquinolone resistance (13 S83 L and D87N, 1 S83A and D87G), although none of the isolates had plasmid-mediated quinolone resistance genes. One isolate had genes expected to confer resistance to almost all antimicrobial classes, including cephalosporins, macrolides, fluoroquinolones, aminoglycosides, and tetracycline, meaning an infection caused by this bacterium would be extremely difficult to treat. A full summary of ARG detections is included in Additional file 2.

### Salmonella

**Fig. 2:**
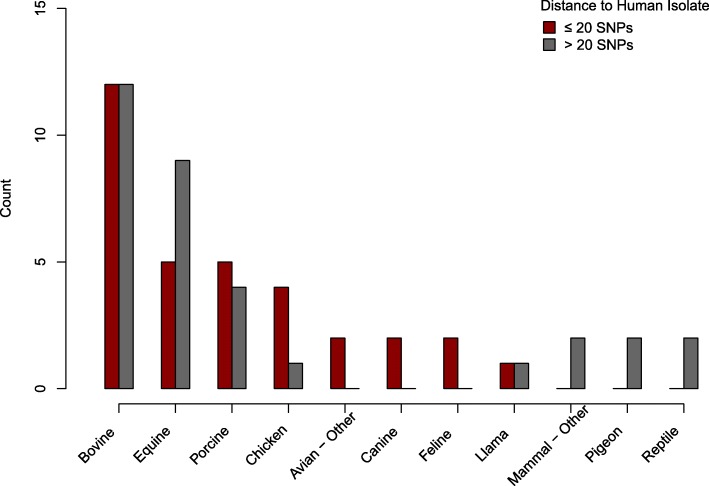
Number of Human-related *S. enterica* Isolates by Host Organism. Red bars show the number of isolates from each host organism that were separated from a human isolate by 20 or fewer SNPs. Grey bars show the number of isolates separated from a human isolate by more than 20 SNPs

**Fig. 3:**
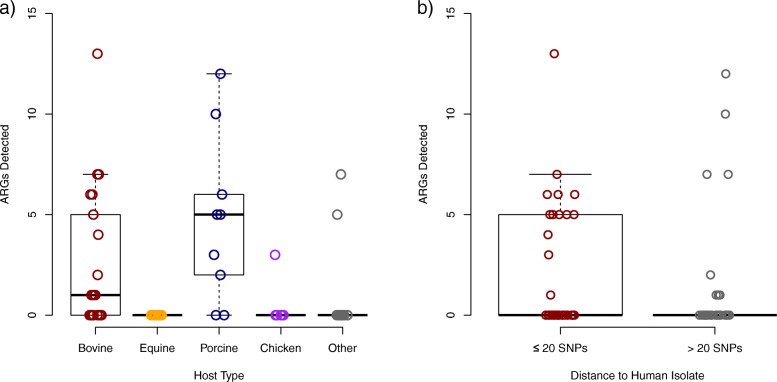
Number of ARGs detected by Host Organism and Human-relatedness. Box-and-whisker plots showing the number of ARGs detected (**a**) in isolates from each host type and (**b**) is isolates separated from a human isolate by 20 or fewer (red) or more than twenty (grey) SNPs

The majority (46/69, 67%) of *Salmonella* had no known resistance genes. The most common host types for *Salmonella* were bovine (*n* = 25), equine (*n* = 15), porcine (*n* = 9) and chicken (*n* = 6). No other host type was shared by more than two isolates. Half (33/66, 50%) of the isolates were separated from a human clinical isolate in the NCBI Pathogen Browser by 20 or fewer SNPs [Fig. 2]. Three isolates were excluded from this analysis because the closest clinical isolate was from a non-human host. The most frequently identified serovar was Typhimurium (*n* = 12), followed by serovars Dublin (*n* = 7) and Newport (*n* = 7) [Table 2]. None of the isolates had fluoroquinolone resistance mutations in *gyrA*. Two isolates had the plasmid-mediated quinolone resistance gene *qnrB5*. These two isolates also had *bla*_CMY-2_ resistance genes, which were present in 10 strains (14%) and confer resistance to cephalosporins and potentiated penicillins. The greatest number of ARGs were detected in porcine and bovine isolates. The only other host types for isolates in which any ARGs were detected were chicken, turkey, and feline [Fig. 3a], and the feline isolate belonged to the bovine-adapted serovar Dublin. It is unclear if these differences in resistance prevalence are broadly representative due to the low number of isolates from each animal source. While the median number of ARGs detected was 0, both for isolates more closely (≤20 SNPs) and distantly (> 20 SNPs) related to human isolates, the distribution skewed towards higher values in the more human-related set, primarily driven by serovar Dublin [Fig. 3b].

**Table 2:** *Salmonella enterica* Serovars

Serovar	Count	Host Type
Typhimurium	12	Bovine (2), Equine (2), Porcine (2), Chicken (2), Pigeon (2), Llama (1), Parrot (1)
Dublin	7	Bovine (6), Feline (1)
Newport	7	Equine (5), Llama (1), Raccoon (1)
Cerro	4	Bovine (3), Chicken (1)
I 4, [5],12:i:-	4	Porcine (2), Equine (1), Canine (1)
Mbandaka	4	Bovine (2), Chicken (1), Canine (1)
Infantis	3	Equine (1), Porcine (1), Feline (1)
Kentucky	3	Equine (1), Chicken (1), Reptile (1)
Braenderup	2	Equine (2)
Derby	2	Porcine (2)
Montevideo	2	Bovine (2)
Uganda	2	Bovine (2)
Other	17	Bovine (7), Equine (4), Porcine (2), Chicken (1), Reptile (1), Turkey (1), Goat (1)

**Fig. 4:**
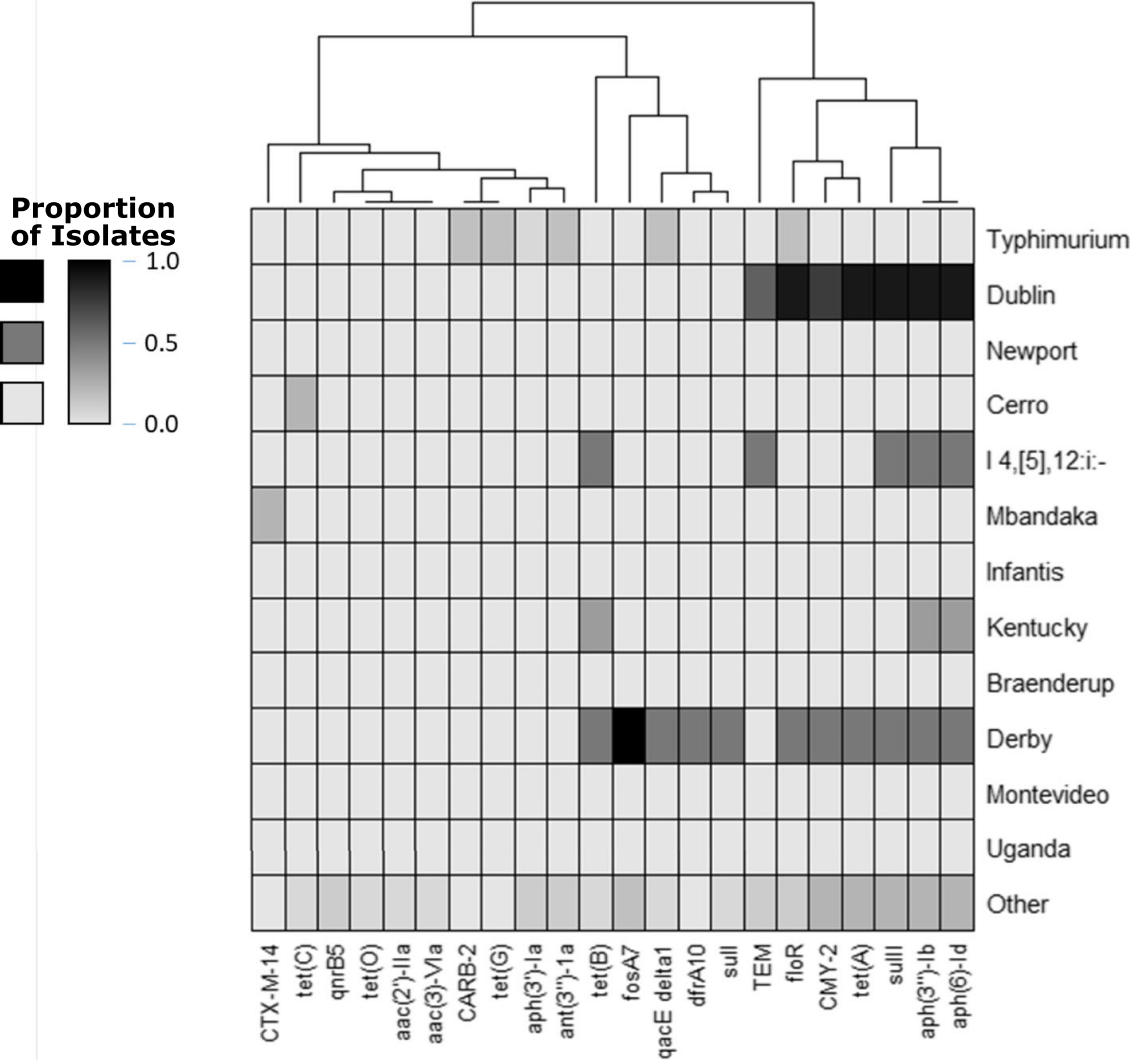
Heatmap of *S. enterica* ARGs by Serovar. Each row corresponds to a serovar, ordered by number of isolates. Each column is an ARG, clustered by co-occurrence as shown by the dendrogram. Darker colors indicate that a given gene is present in a higher proportion of isolates of that serovar

A consistent pattern of ARG presence was found in 6/7 *Salmonella* serovar Dublin isolates, with *sul2, aph(3″)-Ib*/*aph(6)-Id, tet(A),* and *floR* being detected within an approximately 7 kbp window. This pattern was also shared by individual isolates of serovars Agona, Derby, and Heidelberg, suggesting horizontal transfer of this resistance element across distinct lineages [Fig. 4]. These nine isolates were the only ones with an *IncA*/C2 plasmid match detected in PlasmidFinder, with eight also carrying a *bla*_CMY-2_ family beta-lactamase. Together, these genes are predicted to confer resistance to sulfonamides, streptomycin, tetracycline, phenicols, penicillins, and cephalosporins. In assemblies of 3 isolates, two of serovar Dublin and one of serovar Agona, *bla*_CMY-2_ was located on the same contig as the *sul2*–*floR* region. In the serovar Dublin assemblies, it was 28.3 kbp upstream of *sul2* while in the serovar Agona assembly this distance was 29.5 kbp. A full summary of ARG and plasmid detections is included in Additional file 2.

### Staphylococcus pseudintermedius

**Fig. 5:**
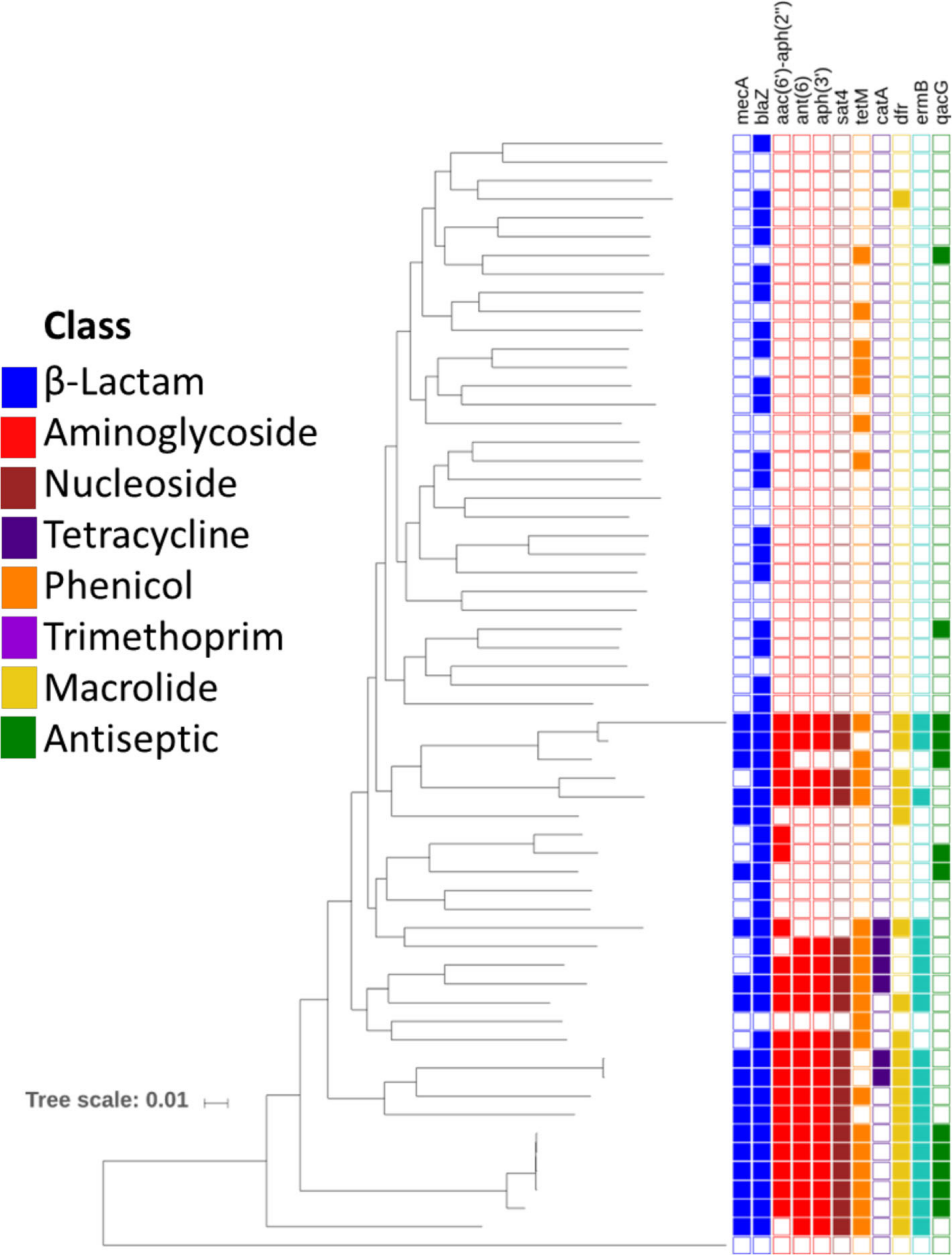
Phylogeny and Antimicrobial Resistance Gene Predictions in *S. pseudintermedius.* Midpoint-rooted core genome phylogenetic tree of *S. pseudintermedius* isolates with ARG predictions. Each column corresponds to the ARG listed along the top, with colors corresponding to the antibiotic class to which that gene confers resistance. A filled box indicates the detection of that gene

The distribution of ARGs per isolate in *S. pseudintermedius* was bimodal, with two or fewer ARGs detected in 36/60 (60%) assemblies and seven or more ARGs were detected in 20/60 (33%) [Fig. 5]. The most frequently detected ARG was a *blaZ* family beta-lactamase, found in 46/60 (77%) assemblies. The tetracycline resistance gene *tetM* was found in 25/60 (42%) and the bifunctional gentamicin/kanamycin resistance gene *aac(6′)-Ie*/*aph(2″)-Ia* was found in 21/60 (35%). The *mecA* gene, which confers methicillin resistance, was detected in 19 isolates comprising 14 different MLST profiles [17]. Nineteen isolates also contained a 2.3 kbp resistance region consisting of *aph*(3′)-IIIa, *sat4*, and an*t(6)-Ia*, which are predicted to confer resistance to kanamycin, streptothricin, and streptomycin, respectively. A *gyrA* S84 L fluoroquinolone resistance mutation was present in 16/60 (27%) isolates. A full summary of ARG detections is included in Additional file 2.

## Discussion

Antimicrobial resistance is a major public health issue of growing importance, which requires comprehensive One Health surveillance and action plans to identify and appropriately respond to the problem. This study fills one important gap in existing AMR surveillance in the U.S. by incorporating bacteria collected from veterinary diagnostic laboratories. By making all of the WGS data publicly accessible, this study also facilitates international research and surveillance efforts.

The majority of *Salmonella* isolates in our study had no ARGs detected, consistent with NARMS human data where approximately 76% of *Salmonella* remain susceptible to all 14 antibiotics on their panel [3]. However, the distribution of ARGs varied across different host types. This could arise from a combination of factors, including variation in the host range of different serotypes, the clinical conditions leading to capture by our sampling framework for different species, and differences in antibiotic exposure. While our data are insufficient to formally assess whether certain host species are more likely to contribute to human infection, they do suggest that companion animals warrant further attention. Serotype-specific differences in resistance prevalence were also not surprising, as some *Salmonella* serovars are known to commonly possess multidrug-resistance elements. Our *Salmonella* Dublin isolates provide an example of the connection between humans, livestock, and companion animals. As would be expected given that it is a cattle-adapted serotype, most of our *Salmonella* Dublin isolates were from bovine hosts. However, the *Salmonella* Dublin isolate with the closest genetic link to a human isolate was from a cat. Human infections with *Salmonella* Dublin are typically associated with exposure to beef and dairy products, exposures which may be shared with companion animals [18]. While clinical history of this cat is unknown, and the source of its infection cannot be determined, we hope continued surveillance of companion animals will enable us to better understand their role in zoonotic transmission pathways as an integral component of the One Health framework.

As with *Salmonella*, most of the *E. coli* isolates from this study did not carry any ARGs. However, we identified one *E. coli* isolate with resistance mechanisms to all major antimicrobial classes, something which has not been observed in NARMS sampling of food animals and retail meats. We also found several *E. coli* isolates with the extended-spectrum beta-lactamase genes *bla*_CTX-M-14_ and *bla*_CTX-M-15_, which were also the most common resistance genes found among isolates from *E. coli* isolated from retail meats and food animals [19]. This suggests the potential relatedness of these strains or their mobile resistance elements, and the higher prevalence of these genes in dog isolates may be in line with previous work that has found pet ownership associated with human colonization of *E. coli* carrying such resistance mechanisms [20].

The *mecA* methicillin resistance gene was detected in 32% of our *S. pseudintermedius* isolates. Methicillin-resistant *S. pseudintermedius* (MRSP) emerged as a significant concern in the early 2000s, with one study finding that the frequency of methicillin resistance among canine *S. pseudintermedius* isolates tested at a veterinary teaching hospital increased from < 5% in 2001 to nearly 30% in 2008 [21]. The overall prevalence of MRSP has been reported from 0 to 4.5% in healthy dogs and up to 7% in dogs with inflammatory skin disease in North America and Europe, with even higher prevalence in some clinical populations [17, 22]. Fourteen distinct MLST profiles were observed amongst *mecA*-positive isolates, indicating that the MRSP population in North America may be more diverse than has previously been reported [23, 24]. In line with previous studies, we also found that *mecA*-positive isolates tended to carry genes expected to confer resistance to multiple other classes of antibiotics, limiting treatment options [9, 10, 17, 23, 25, 26]. While there is evidence of *S. pseudintermedius* strain sharing between pets and their owners, the extent to which contact with companion animals increases risk is unclear [9, 10, 27].

Together, these data underscore the relevance of AMR monitoring of bacteria causing significant disease in animal species from veterinary diagnostic labs, fulfilling our objectives to establish an animal AMR monitoring system. This component should not be overlooked as part of any One Health national surveillance strategy, and Vet-LIRN will continue to monitor resistance in *Salmonella*, *E. coli*, and *S. pseudintermedius*, providing important information on temporal trends. These data will be used to design further surveillance studies and to supplement data from existing surveillance programs as we strive to develop evidence-based practices to support the reduction of AMR in human and animal pathogens.

## Conclusion

This study highlights the utility of performing AMR surveillance of bacteria from veterinary diagnostic laboratories as a part of any national surveillance program. The incorporation of companion animals helps address a key gap in the current national AMR surveillance framework as part of a One Health paradigm. As the isolates for this study were obtained from clinical cases submitted for diagnosis, we acknowledge that they are not representative the overall population of these bacteria in targeted host species. However, they can serve as a valuable sentinel population, as shown by the finding of some highly resistant bacterial strains, including some related to those from humans. WGS has become a crucial tool to identify the origins and spread of AMR and to develop successful One Health surveillance strategies. Such surveillance studies will help to assess trends in AMR over time and can facilitate the development of public policies based on sound science. Vet-LIRN will continue to support our laboratories’ participation in AMR monitoring of veterinary pathogens as part of fulfilling its mission of advancing human and animal health.

## Methods

### Pathogen selection

Vet-LIRN selected two microbial pathogens monitored by NARMS: *Salmonella enterica* and *Escherichia coli*. A third pathogen, *Staphylococcus pseudintermedius*, was selected based on the results of the Working Group survey. Isolates of *Salmonella* were collected from all animal hosts, and *E. coli* and *S. pseudintermedius* isolates were collected only from dogs, with all bacteria being derived from clinically sick animals.

### Participating laboratories and planned isolate collection

**Fig. 6:**
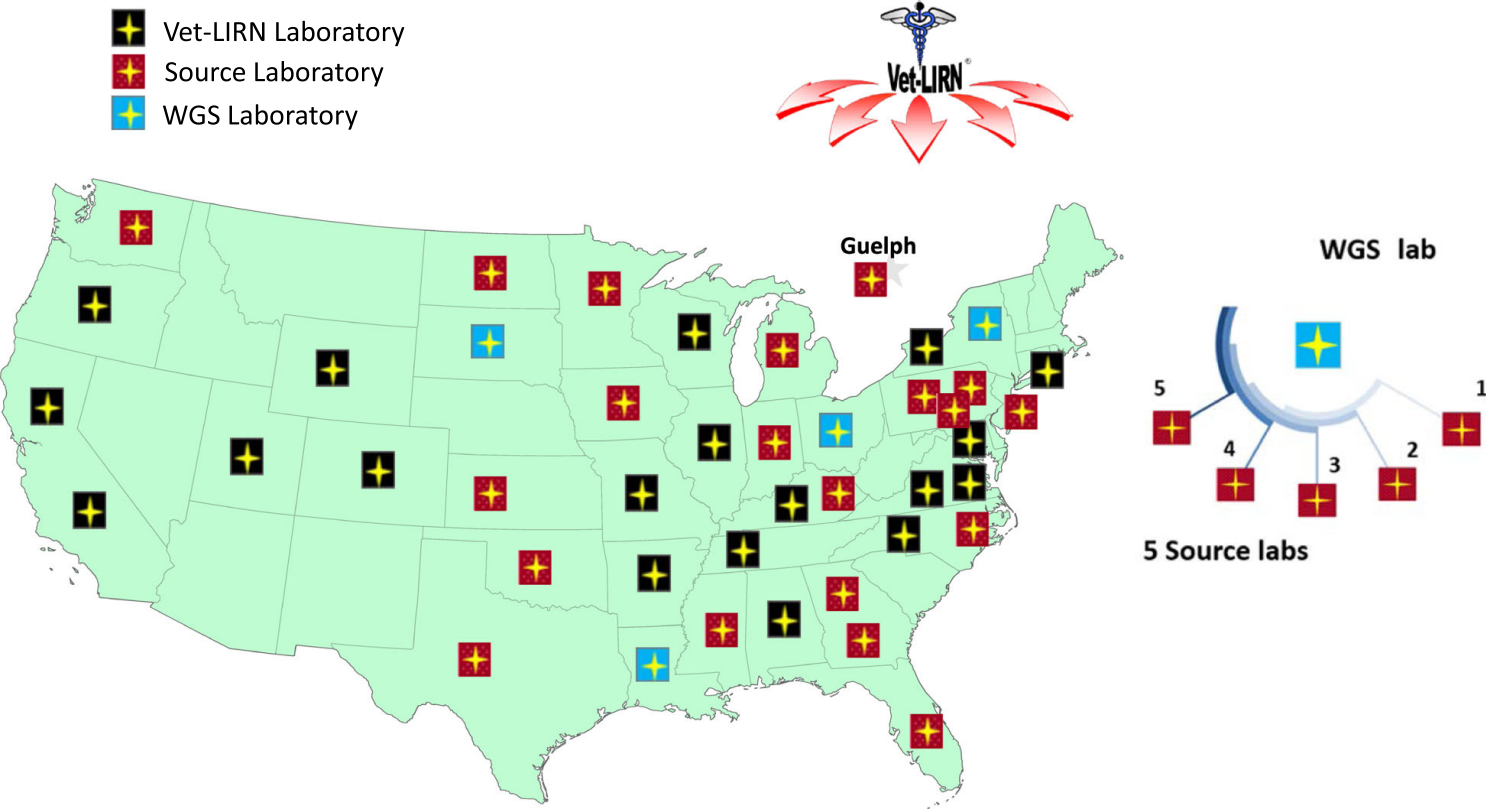
Geographical distribution and organization of Vet-LIRN WGS and Source laboratories. Twenty source laboratories (19 is the U.S. and one in Canada) (red) collected isolates. Four WGS labs (blue) selected five collaborating source labs each and sequenced a subset of the isolates submitted by their source labs. Remaining Vet-LIRN laboratories, currently not participating in the project, are shown in black. Additional labs became source labs in 2018. License for using and editing US Map Template for Power Point was purchased from Envato Pty Ltd., PO Box 16,122, Collins Street West, Victoria, 8007 Australia

Isolates were collected by a network of 20 Vet-LIRN veterinary diagnostic laboratories (“source laboratories”). Each of these were partnered with one of four WGS laboratories. Figure 6 shows the geographical distribution and organization of Vet-LIRN WGS and source laboratories in 2017. All laboratories were affiliated with either an academic institution or U.S. state government. Source laboratories collected the first four isolates each month, from each of the three selected pathogens, *S. enterica*, *E. coli*, and *S. pseudintermedius*, for a potential total of 144 isolates per source laboratory. The potential total number of isolates for 2017 was 2880.

U.S. laboratories serotyped all *Salmonella* isolates either in-house or by referral to the USDA National Veterinary Services Laboratory. Isolates from Canada were serotyped by Public Health Agency of Canada National Microbiology Laboratory. Laboratories were instructed to select only one isolate per client submission. Isolate species were determined by either analytical profile index (API), matrix assisted laser desorption/ionization-time of flight (MALDI-TOF) mass spectrometry, polymerase chain reaction (PCR), Sensititre, Vitek, or biochemical identification. A frozen aliquot of each isolate was sent to the corresponding WGS laboratory. Each quarter, Vet-LIRN randomly selected one isolate of each pathogen species from each source laboratory to be sequenced.

Source laboratories submitted metadata for each isolate, while anonymizing certain features by omitting specific geographic location and client information. In the U.S., veterinarians are required by the Principles of Veterinary Medical Ethics [28], and by law [29], to keep the medical records of their patients confidential. Metadata was collected using the metadata sheet developed by the GenomeTrakr program [30], with additional information required by the Vet-LIRN Program Office. Those fields included the information on which source lab collected the isolate, Vet-LIRN specific isolate ID, isolate taxonomic name, date of collection (day, month, or year), U.S. state, specific animal host, case type (primary, secondary, tertiary), as well as the anatomical site from which the pathogen was isolated. A complete metadata sheet template is provided as Additional file 1.

Four sequencing laboratories (“WGS labs”) each had five collaborating source labs (Fig. 6) and sequenced a subset of the isolates submitted by their source labs quarterly. These isolates were selected at random by the Vet-LIRN program office, to obtain a snapshot of the pathogens being cultured at referral veterinary laboratories. One isolate of each pathogen species was sequenced per quarter, from each of the source labs. Depending on the case load of source labs, each WGS lab was expected to sequence up to 60 isolates/year, for a potential total of 240 isolates for all of 2017.

### Whole genome sequencing (WGS)

After harmonizing the test method across four different laboratories and passing an FDA GenomeTrakr program proficiency test, Vet-LIRN WGS laboratories sequenced the isolates. DNA was extracted from either a single colony, or a pellet of a liquid culture from a single colony, using the DNeasy Blood and Tissue Kit (QIAGEN Sciences, Germantown, MD). DNA quality control was performed using Qubit instrumentation and reagents (Thermo Fisher Scientific, Waltham, MA). Genomic libraries were prepared following the Nextera XT Library Preparation Kit protocol (Illumina, Inc.) according to the manufacturer’s instructions. Laboratories had the option of normalizing libraries either using the Illumina bead-based normalization procedure or by concentration of the purified libraries using Qubit. Sequencing was performed on the Illumina MiSeq platform using v2, 2x250bp chemistry (Illumina, Inc., San Diego, CA).

### Sequence analysis

All sequencing reads were uploaded to National Center for Biotechnology Information (NCBI) SRA under BioProjects PRJNA316449, PRJNA314607, and PRJNA316451. Isolate-level accession numbers are listed in Additional file 2. Any samples with an average coverage of less than 30X were repeated until they met this threshold. Low quality segments were removed using the Trimmomatic version 0.36 sliding window program with a window size of 4 and minimum quality score of 20 [31]. Trimmed reads were then assembled using SPAdes version 3.10.1 [32]. Assembly quality was assessed using Quast version 4.0 [33] and contigs were classified using Kraken2 [34]. Samples were excluded from further analysis if they showed evidence of substantial contamination.

Parsnp was used to generate a core genome alignment phylogenetic tree for each of the three species [35]. Assemblies were screened for AMR genes in the NCBI and ARG-ANNOT [36] databases and plasmids in the PlasmidFinder [37] database using ABRicate version 0.8 (https://github.com/tseemann/abricate). Endogenous and ubiquitously detected resistance genes (*ampC, ampH*, and penicillin-binding protein in *E. coli*) and regulatory genes (*tetR* in *S. enterica* and *E. coli* and *mecI* and *mecR1* in *S. pseudintermedius*) were excluded from antimicrobial resistance gene (ARG) counts but are listed in Additional file 2. Trees and ARG predictions were visualized using iTOL [38]. Assemblies were annotated using Prokka [39], and the *gyrA* gene was searched for amino acid changes associated with fluoroquinolone resistance: amino acids 83 and 87 in *E. coli* and *Salmonella* and 84 in *S. pseudintermedius* [25, 40, 41]. *Salmonella* serovar predictions were generated using SISTR version 1.0.2 [42]. *E. coli* phylogroups were determined by searching each assembly for the Clermont quadriplex PCR primers using BLAST [43] and verifying that they would produce a PCR product of the expected size [44]. Ambiguous phylogroup predictions were verified by comparing to the core genome phylogeny and manually examining the target sequence fragments. Multilocus sequence typing (MLST) profiles were determined using SRST2 and the seven-locus *S. pseudintermedius* MLST scheme hosted on PubMLST (https://pubmlst.org/spseudintermedius/, accessed October 31, 2018) [24, 45]. For *Salmonella* isolates, we obtained the SNP distance to the nearest clinical isolate, assumed to be of human origin, using the NCBI Pathogen Detection Isolate Browser (https://www.ncbi.nlm.nih.gov/pathogens/, accessed February 21, 2019) [46]. Isolates were excluded from SNP distance comparisons if the nearest clinical isolate was specified as originating from a non-human host. A distance of ≤20 SNPs was used as a threshold for potential relatedness [47].

## Additional files

Additional file 1: Metadata entry template sheet. (XLSX 284 kb)

Additional file 2: A table with full summary of ARG and plasmid detections and NCBI isolate-level accession numbers. (XLSX 33 kb)

## Abbreviations

AAVLD: American Association of Veterinary Laboratory Diagnosticians
AMR: Antimicrobial resistance
API: Analytical profile index
ARGs: antimicrobial resistance genes
AST: Antibiotic susceptibility testing
CARB: Combating Antibiotic-Resistant Bacteria
CDC: Centers for Disease Control and Prevention
CLSI: Clinical and Laboratory Standards Institute.
CVM: Center for Veterinary Medicine
FDA: Food and Drug Administration
MALDI-TOF: Matrix Assisted Laser Desorption/Ionization-Time of Flight
NAHLN: National Animal Health Laboratory Network
NARMS: National Antimicrobial Resistance Monitoring System
NCBI: National Center for Biotechnology Information
PCR: Polymerase chain reaction
USDA: United States Department of Agriculture
Vet-LIRN: Veterinary Laboratory Investigation and Response Network
WGS: Whole genome sequencing
Working Group: AAVLD Antimicrobial Resistance Working Group
